# Mitral regurgitation severity dynamic during acute decompensated heart failure treatment

**DOI:** 10.1007/s10554-021-02495-7

**Published:** 2021-12-22

**Authors:** Kamil Bugała, Paweł Rubiś, Mateusz K. Hołda, Małgorzata Konieczyńska, Piotr Bijak, Wojciech Płazak

**Affiliations:** 1 Department of Diagnostics, John Paul II Hospital, Kraków, Poland; 2 Department of Cardiac and Vascular Diseases, Jagiellonian University Medical College, John Paul II Hospital in Kraków, ul. Prądnicka 80, 31-202 Kraków, Poland; 3 HEART – Heart Embryology and Anatomy Research Team, Department of Anatomy, Jagiellonian University Medical College, Kraków, Poland; 4 Department of Cardiovascular Sciences, University of Manchester, Manchester, UK

**Keywords:** Heart failure, Mitral valve, Mitral regurgitation, Echocardiography

## Abstract

Acute decompensated heart failure (ADHF) treatment leads to significant hemodynamic changes. The aim of our study was to quantitatively analyze the dynamics of mitral regurgitation (MR) severity (evaluated by transthoracic echocardiography) which occur during the treatment of ADHF and to correlate these changes with the clinical condition of patients as well as heart failure biochemical markers. The study included 27 consecutive adult patients (40.7% females, mean age 71.19 ± 11.2 years) who required hospitalization due to signs of acute HF. Echocardiographic assessment was performed upon admission and discharge together with clinical and laboratory evaluation. Significant reduction in dyspnea intensity [0–100 scale] (81.48 ± 9.07 vs. 45.00 ± 11.04 pts, p < 0.001), body weight (84.98 ± 18.52 vs. 79.77 ± 17.49 kg, p < 0.001), and NT-proBNP level (7520.56 ± 5288.62 vs. 4949.88 ± 3687.86 pg/ml, p = 0.001) was found. The severity of MR parameters decreased significantly (MR volume 44.92 ± 22.83 vs. 30.88 ± 18.77 ml, p < 0.001; EROA 0.37 ± 0.17 vs. 0.25 ± 0.16 cm^2^, p < 0.001; VC 6.21 ± 1.48 vs. 5.26 ± 1.61 mm, p < 0.001). Left atrial area (35.86 ± 9.11 vs. 32.47 ± 9.37, p < 0.001) and mitral annular diameter (42.33 ± 6.63 vs. 39.72 ± 5.05. p < 0.001) also underwent statistically significant reductions. An increase in LVEF was observed (34.73 ± 13.88 vs. 40.24 ± 13.19%, p < 0.001). In 40.7% of patients, a change in MR severity class (transition from a higher class to a lower one) was observed: 6/8 (75%) patients transitioned from severe to moderate and 6/18 (33.3%) patients transitioned from moderate to mild class. Treatment of ADHF leads to a significant reduction in MR severity, together with significant reductions in left atrial and mitral annular dimensions. Quantitative measurement of MR dynamics offer valuable assistance for ADHF management.

## Introduction

Acute decompensated heart failure (ADHF) is defined as a sudden worsening of the signs and symptoms of heart failure, and is often a potentially life-threatening condition requiring hospitalization and emergency therapy [1]. Long-term, optimal treatment of heart failure significantly improves patient prognosis by reversing the unfavorable remodeling of the left ventricular myocardium [2].

Mitral valve regurgitation is frequently observed in patients hospitalized for ADHF and is associated with significant dilatation of the left ventricular cavity and mitral annular distension. Severe mitral valve insufficiency diagnosed on admission due to ADHF symptoms is associated with an unfavorable prognosis [3]. Pharmacotherapy used in ADHF treatment has a significant impact on the dynamics of mitral valve insufficiency [4–6]. It has been shown that optimal treatment of acute heart failure, using diuretics and vasodilators, leads to reductions in left ventricular filling pressure and systemic vascular resistance, and thus, significantly reduces mitral regurgitation [4]. Moreover, it has been demonstrated that this phenomenon occurs mainly in the mechanism of mitral ring diameter reduction and decreased left ventricular end-diastolic dimension, both of which lead to a reduction in the regurgitant orifice area [7].

Because the severity of mitral regurgitation may significantly change during pharmacological treatment of ADHF, mitral valve evaluation by echocardiographic imaging could have a significant impact on the qualification of patients for surgical management of mitral valve disease. Nevertheless, there are only a few studies describing the changes in cardiac hemodynamics during ADHF management and the impact of these changes on long-term prognosis [8]. Therefore, the aim of our study was to quantitatively analyze changes in mitral regurgitation severity, as evaluated by transthoracic echocardiography, which occur during the treatment of ADHF and to correlate these changes with the clinical condition of patients as well as with heart failure biochemical markers.

## Materials and methods

### Study population

The study included 27 consecutive adult patients (40.7% females, mean age 71.19 ± 11.2 years) requiring hospitalization due to signs and symptoms of acute heart failure and were admitted to the Department of Cardiac and Vascular Diseases and Department of Diagnostics, Jagiellonian University Medical College, John Paul II Hospital in Kraków, Poland. Exclusion criteria were: fever, infection or sepsis upon admission, confirmed diagnosis of concomitant acute coronary syndrome, acute heart failure caused by significant cardiac arrhythmias (tachycardia or bradycardia), hyperthyroidism/hypothyroidism, pregnancy and puerperium.

### Patient management and evaluation

All enrolled patients received conventional pharmacological treatment in accordance with current guidelines for the treatment of acute heart failure [9]. Inclusion into the study did not result in disruption of therapy, which was determined solely by the attending physician.

The standard diagnostic scheme was used in all patients, which included an assessment of basic vital sign parameters (heart and respiration rate, peripheral oxygen saturation and arterial blood pressure). Resting electrocardiogram was performed upon admission. Body mass and fluid balance was monitored daily. Basic biochemical tests were performed. Additionally, serum NT-proBNP level was determined on the first day of treatment and upon discharge. A subjective assessment of clinical symptoms (severity of dyspnea) was also performed using a scale from 0 to 100 points.

### Echocardiographic assessment

Per-protocol transthoracic echocardiography was performed in all patients on admission and on the day of discharge. All examinations were performed by one researcher with several years of experience in performing echocardiographic examinations using the Philips iE33 and Philips Epiq ultrasound machines (Philips Healthcare, Eindhoven, Netherlands). Additionally all examinations were supervised by the head of Echocardiography Department. Post-processing and study evaluation were performed using a dedicated workstation. All linear measurements were performed using virtual calipers. Echocardiographic assessment of heart structure and mitral regurgitation was performed according to current guidelines [10].

The following parameters were analyzed: left ventricular end-diastolic (LVEDD) and end-systolic (LVESD) dimensions; left ventricular end-diastolic (LVEDV) and end-systolic (LVESV) volume; left ventricular ejection fraction (LVEF, calculated using the Simpson biplane method); left atrial dimension (measured in the parasternal long axis view); left atrial surface (measured in the apical four-chamber view); mitral annular diameters (measured in the parasternal long axis and apical four-chamber views); mitral inflow parameters (peak E-wave and A-wave velocity, E/A ratio). In cases of atrial fibrillation, measurements from 3 to 5 heart rates were obtained and mean values were calculated. The assessment of mitral regurgitation severity was carried out based on the following parameters: semi-quantitative assessment using Vena Contracta (VC) measurement; quantitative assessment using the flow convergence (FC) method; Proximal Isovelocity Surface Area (PISA) radius; effective regurgitant orifice area (EROA); and mitral regurgitant volume (MR Vol).

### Statistical analysis

Data are presented as mean ± standard deviation (SD) with the median and interquartile ranges (Q1, Q3) for continuous variables or percentages (%) for categorical variables. Distribution analysis of the studied data was performed using the Kolmogorov–Smirnov test. The Student’s t-test and Mann–Whitney U test were used for statistical comparisons. A p-value < 0.05 was considered statistically significant. Statistical analyses were performed using StatSoft STATISTICA 13.1 software (StatSoft Inc., Tulsa, OK, USA).

This study was approved by the Bioethical Committee of the Jagiellonian University in Kraków, Poland (No 122.6120.294.2015). The study protocol conforms to the ethical guidelines of the 1975 Declaration of Helsinki. Written informed consent was obtained from all patients.

## Results

All enrolled patients initially presented with symptoms of significant dyspnea (either NYHA class III or IV). Ischemic etiology of heart failure was found in 48% of patients. The average subjective severity of dyspnea (assessed on a 0–100 point scale) was 81.5 ± 9.07 pts. Detailed clinical characteristics of patients on admission are presented in Table 1. Most of the included patients had previously received standard treatment for chronic heart failure. Angiotensin-converting enzyme inhibitors or aldosterone receptor blockers were used in 70.4% of patients, while all patients were chronically treated with beta-blockers (Table 1). Seven patients from the study population required treatment with vasopressors and inotropes, while none required the use of an intra-aortic balloon pump or mechanical ventilation. The level of NT-proBNP was high in all patients with a mean value of 7520.0 ± 5645.25 pg/ml. Atrial fibrillation was noted in 74.1% of patients, including persistent or permanent atrial fibrillation in 5.4% of patients. No significant differences in terms of patient sex were found in any of the baseline clinical parameters. The average daily dosages of administrated diuretics, ACE-I, MRA and beta-blockers are presented in Table 2. During ADHF treatment, patient body mass and NT-proBNP level decreased significantly, together with a significant decrease in dyspnea (Table 3).

**Table 1:** Clinical characteristics of the study group on admission (n = 27)

Clinical parameter	Value at baseline
Age (years)	71.2 ± 11.2 (range 41.0–90.0)
BMI	30.2 ± 5.4 (range 22.2–41.2)
QRS duration (ms)	116.2 ± 27.0 (range 80.0–160.0)
Arterial hypertension (%)	92.6
Diabetes mellitus (%)	37.0
Atrial fibrillation (%)	74.1
COPD (%)	7.4
Coronary artery disease (%)	48.1
Previous myocardial infarction (%)	37.0
Previous CABG/PCI (%)	40.7
LBBB in ECG (%)	15.4
RBBB in ECG (%)	11.5
Chronic treatment with ACE-I (%)	70.4
Chronic treatment with beta-blockers (%)	100
Chronic treatment with MRA (%)	70.4

Data are expressed as the mean value ± standard deviation and range for continuous variables and as a percentage of patients for qualitative variables

*ACE-I *angiotensin-converting enzyme inhibitors, *BMI* ody mass index, *CABG* oronary artery bypass grafting, *COPD* chronic obstructive pulmonary disease, *MRA* aldosterone receptor antagonists, *PCI* percutaneous coronary intervention

**Table 2:** Mean daily doses of administrated diuretics, beta-blockers, ACEI and MRA

		At admission	At discharge
Beta-blockers (mg/24 h)	Metoprolol succinate	43.75 ± 11.57	40.62 ± 12.93
	Bisoprolol	3.36 ± 2.41	4.46 ± 2.97
	Carvedilol	14.58 ± 9.54	12.5 ± 10.82
	Nebivolol	3.75 ± 1.76	1.87 ± 0.88
ACEI (mg/24 h)	Perindopril	3.75 ± 1.76	3.75 ± 1.76
	Ramipril	4.68 ± 2.86	3.21 ± 1.44
MRA (mg/24 h)	Spironolactone	31.66 ± 11.44	37.5 ± 13.17
	Eplerenone	37.5 ± 14.43	34.72 ± 15.02
Diuretics (mg/24 h)	Furosemide	101.48 ± 79.40	90.40 ± 46.23
	Torasemide	8.70 ± 13.97	25.19 ± 41.19

Data are expressed as the mean value ± standard deviation

*ACEI* angiotensin-converting enzyme inhibitors, *MRA* aldosterone receptor antagonists

**Table 3:** Change in heart failure parameters during the treatment period

Parameter	Value at baseline	Value at discharge	Value change (baseline-discharge)	p-value
Level of dyspnea (0–100 pts)	81.48 ± 9.07 (range 60–90)	45 ± 11.04	36.54	< 0.001
Body weight (kg)	84.98 ± 18.52	79.77 ± 17.49	5.21	< 0.001
NT-proBNP (pg/ml)	7520.56 ± 5288.62	4949.88 ± 3687.86	2570.68	0.001

Data are expressed as the mean value ± standard deviation

Echocardiographic parameters are presented in Table 4. Initially, patients showed significant remodeling of the left ventricular myocardium (mean LVEDV = 179.1 ± 80.51 ml) and significant dilatation of the mitral valve annulus (mean 42.3 ± 6.63 mm). Moreover, left ventricular systolic function was significantly reduced (mean LVEF = 34.73 ± 13.88%). Mitral regurgitation was assessed as severe in 29.6% of patients. In our patients mitral regurgitation was connected to left ventricular remodeling and mitral annulus dilation.

**Table 4:** Change in echocardiographic parameters during the treatment period (n = 27)

Parameter	Value at baseline	Value at discharge	Value change (baseline-discharge)	P-value
LA diameter PLAX (mm)	54.81 ± 6.57	50.77 ± 6.32	4.04	< 0.001
LA area in AP4 (cm^2^)	35.86 ± 9.11	32.47 ± 9.37	3.38	< 0.001
LVEDD in PLAX (mm)	61± 11.41	58.35 ± 11.03	2.65	< 0.001
LVESD in PLAX (mm)	48.35 ± 13.47	46.65 ± 13.27	1.69	0.002
LVEDV (Simpson biplane method) (ml)	179.08 ± 80.51	174.15 ± 80.03	4.92	0.307
LVEF (Simpson biplane method) (%)	34.73 ± 13.88	40.24	5.50	< 0.001
Mitral annular diameter in PLAX (mm)	42.33 ± 6.63	39.72 ± 5.05	2.61	< 0.001
Mitral annular diameter in AP4 (mm)	42.46 ± 4.99	39.67 ± 5.13	2.79	< 0.001
Mitral mean gradient (mmHg)	2.25 ±1.31	1.81 ± 0.89	0.45	0.02
Peak E-wave velocity (cm/s)	122.83 ± 31.29	107.33 ± 23.61	15.50	< 0.001
E/A ratio	0.95 ± 1.22	0.94 ± 1.16	0.004	0.98
VC (mm)	6.21 ± 1.48	5.26 ± 1.61	0.95	< 0.001
PISA MR radius (mm)	7.84 ± 2.65	6.36 ± 2.25	1.04	< 0.001
MR VTI (cm)	136.32 ± 24.67	136.37 ± 23.84	-0.05	0.98
MR V max (m/s)	4.61 ± 0.63	4.53 ± 0.65	0.07	0.4
MR EROA (cm^2^)	0.37 ± 0.17	0.25 ± 0.16	0.11	< 0.001
MR volume (ml)	44.92 ± 22.83	30.88 ± 18.77	14.04	< 0.001

Data are expressed as the mean value ± standard deviation

*AP4* apical four chambers view EROA-effective regurgitant orifice area, *LA* left atrium, *LVEDD* left ventricular end diastolic diameter, *LVEDV* left ventricular end diastolic volume, *LVEF* left ventricular ejection fraction, *LVESD* left ventricular end systolic diameter, *LVESV* left ventricular end systolic volume, *MR* mitral regurgitation, *PISA* proximal Isovelocity Surface Area, *PLAX* parasternal long axis view, *VC* vena contracta, *VTI* velocity time integral

A significant decrease in the severity of mitral regurgitation echocardiographic parameters was observed, especially in MR volume (31% reduction), EROA (32% reduction), and VC (15% reduction) (Table 4). Left atrial and mitral annular dimensions also underwent a statistically significant reduction (Table 4). Left ventricular ejection fraction increased from 34 to 40%. Nevertheless, no significant changes in LVEDV, velocity time integral (MR VTI), or maximum velocity of the mitral return wave (MR Vmax) were noted (Table 4).

**Fig. 1:**
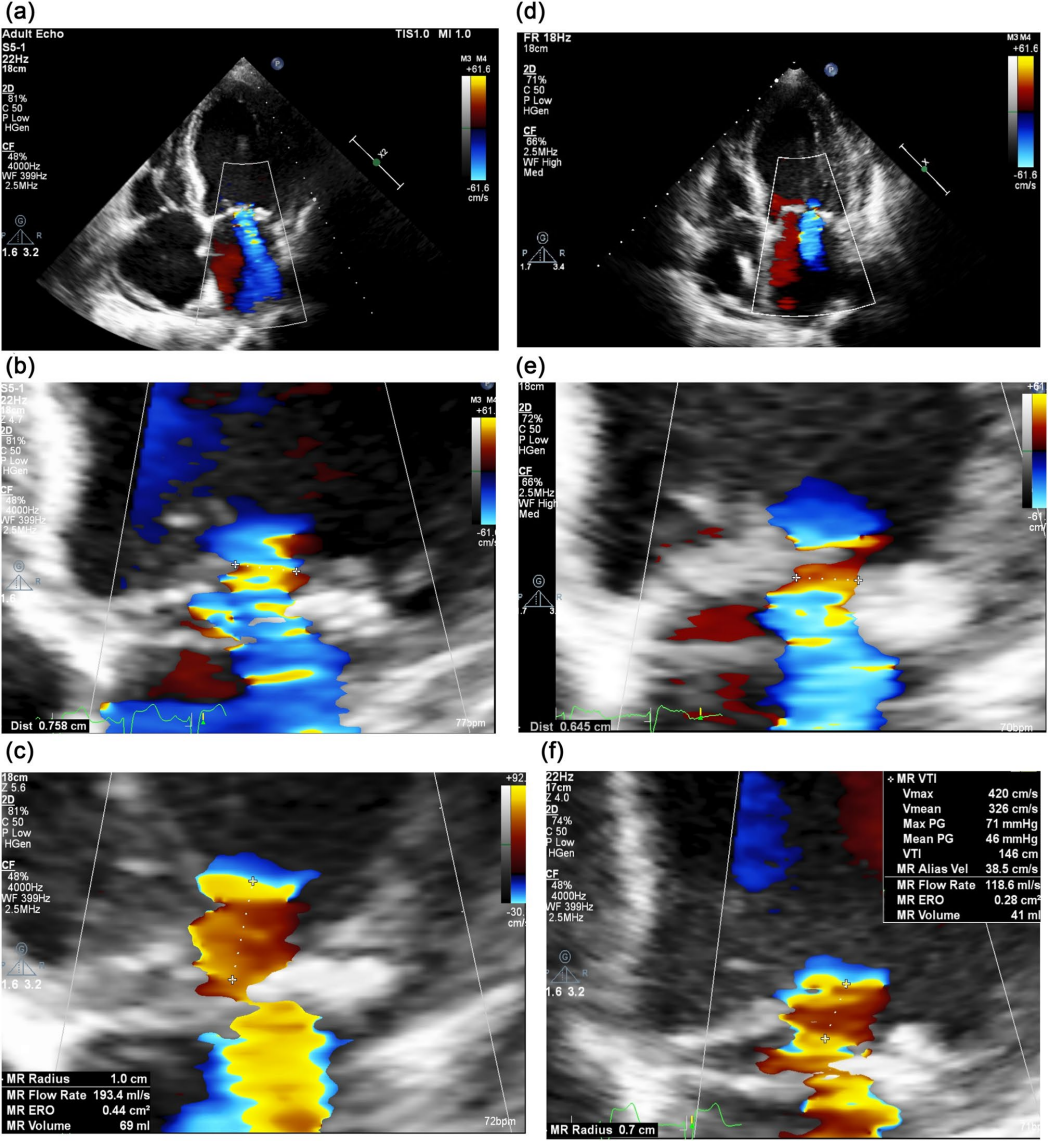
Images showing changes in the severity of mitral regurgitation during the treatment of ADHF. Initially severe mitral regurgitation—**a**, **b** and **c** in echocardiographic assessment on discharge was evaluated as moderate—(**d**), (**e**) and (**f**)

In 40.7% of patients, a change in mitral regurgitation severity (transition from one class to another) was observed during the hospitalization period (Table 5). Specifically, 75.0% of patients with severe mitral regurgitation at baseline had improved to moderate, while 33.3% of patients with an initially moderate wave had improved to mild (Table 5). An exemplary echocardiographic assessment of changes in the severity of mitral regurgitation is presented in the Fig. 1.

**Table 5:** Categorical changes in the severity of mitral regurgitation and a comparison of baseline and discharge number of patients in each group (mild, moderate, severe)

Severity of mitral regurgitation	Baseline n (%)	Discharge n (%)
Mild	1 (3.7)	7 (25.9)
Moderate	18 (66.7)	18 (66.7)
Severe	8 (29.6)	2 (7.4)
Baseline	Discharge	
Mild 1 patient	Mild 1 patient	
	Moderate 0 patients	
	Severe 0 patients	
	Mild 6 patients	
Moderate 18 patients	Moderate 12 patients	
	Severe 0 patients	
	Mild 0 patients	
	Moderate 6 patients	
Severe 8 patient	Severe 2 patients	

n number of patients in each category

## Discussion

The main finding of the present study is to show significant quantitative reduction of mitral regurgitation in patients treated due to ADHF. In 40% of patients, the reduction led to a change in mitral regurgitation severity class, which may be the most important factor in the decision-making process concerning surgical treatment of such patients.

The results of our current study demonstrate the dynamic nature of mitral regurgitation in patients with ADHF. Until now, studies describing mitral regurgitation severity during the treatment of acute cardiovascular decompensation, as assessed by echocardiography, were mainly based on semi-quantitative measurements. In our work, apart from semi-quantitative measurements, results from quantitative assessment of the mitral regurgitation are also presented. The reduction in mitral regurgitation severity during ADHF treatment is associated with a combination of many different factors such as a reduction in afterload, pressure gradient change between the left ventricle and left atrium, and a reduction in left ventricular and mitral annular dimensions, which are associated with a reduction in EROA [7]. In line with previous observations involving groups of patients treated for acute heart failure [7], our study also showed a significant reduction in the dimensions of the left atrium, mitral annular diameter, and EROA, assessed using the PISA method, when comparing baseline measurements with discharge measurements. On the other hand, no significant reduction was recorded in the LVEDV when assessed by the biplanar method. This could be related to the baseline characteristics of the study population, which was affected by advanced left ventricular remodeling at baseline.

The presence of significant ischemic mitral regurgitation in patients with a history of myocardial infarction and heart failure with reduced ejection fraction significantly worsens the long-term prognosis, regardless of the LVEF, age, and NYHA functional class [11, 12]. In contrast to primary mitral regurgitation, there is still no clear evidence whether a reduction in secondary mitral regurgitation is associated with improved survival [13]. Nevertheless, minimally invasive procedures such as the percutaneous edge-to-edge repair system (MitraClip) are commonly used, which can provide a reduction in the severity of heart failure symptoms and are associated with a reduction in unfavorable remodeling of the left ventricular myocardium [14]. As shown in the COAPT trial, the appropriate selection of heart failure patients for the MitraClip procedure leads to a reduction in the number of hospitalizations and decreased mortality in the 24-month follow-up [15].

Our study illustrates the importance of a solid understanding of mitral regurgitation dynamics in heart failure patients in everyday clinical practice. Importantly, our study showed a significant reduction in mitral regurgitation severity (8 patients with severe regurgitation at baseline vs. 2 patients at discharge) as a result of standard treatment for cardiovascular decompensation. This may have a significant impact on the selection of management strategies in patients, including the decision to perform surgical treatment for valvular disease. In chronic severe secondary mitral regurgitation (SMR) valve surgery is often associated with high risk of complications. Indications for isolated valve surgery are of course restrictive. We consider surgery in patient with concomitant coronary artery disease who are candidates for CABG. In other cases of SMR persisting severe despite optimal treatment (including CRT if indicated) we can consider transcatheter edge-to-edge repair. Quantification of functional mitral regurgitation should always be performed after stabilization of heart failure decompensation and reduction of fluid overload.

It is also worth mentioning that in the group of patients with initially severe mitral regurgitation admitted to the hospital due to symptoms of ADHF, mitral regurgitation reduction to moderate or mild classes do not necessarily imply an improved prognosis. This was demonstrated in the group of patients with ADHF and dynamic mitral regurgitation initially meeting the criteria of a severe wave, which then significantly reduces during treatment process, where the prognosis is much worse than in the patients with insignificant regurgitation, but at a similar level as in the group of patients with persistent severe mitral regurgitation [3]. Therefore, patients with dynamic and severe mitral regurgitation also require greater attention and further follow-up to introduce a suitable treatment, including surgical management if needed.

## Limitations of the study

A limitation of our study is the fact that it is a single-center analysis with a relatively small study group, involving patients diagnosed with ischemic (48%) and non-ischemic (52%) heart dysfunction. However, we believe that due to the high statistical significance of the observed changes, this does not interfere with our quantitative, qualitative, and clinical analyses of mitral regurgitation associated with ADHF.

## Conclusions

Standard pharmacological treatment in ADHF patients leads to a significant reduction in mitral valve severity expressed as a reduction in mitral regurgitation volume, EROA, and vena contracta width. As a result of treatment, most of the patients with severe mitral regurgitation were then classified as having moderate mitral regurgitation. A significant reduction in left atrial and mitral annular dimensions were also observed. Quantitative measurement of mitral regurgitation dynamics could offer valuable information for the management of ADHF patients. Observed changes of mitral insufficiency may also serve for future studies evaluating length of the treatment of ADHF and tailoring the therapy to the individual patient.

## Data Availability

Data are available to researchers on request for purposes of reproducing the results by directly contacting the corresponding author.
